# Anti-Inflammatory, Antinociceptive, Antipyretic, and Gastroprotective Effects of *Eurycoma longifolia* Jack Ethanolic Extract

**DOI:** 10.3390/life13071465

**Published:** 2023-06-28

**Authors:** Subhawat Subhawa, Warangkana Arpornchayanon, Kanjana Jaijoy, Sunee Chansakaow, Noppamas Soonthornchareonnon, Seewaboon Sireeratawong

**Affiliations:** 1 Clinical Research Center for Food and Herbal Product Trials and Development (CR-FAH), Faculty of Medicine, Chiang Mai University, Chiang Mai 50200, Thailand; subhawat.s@cmu.ac.th; 2 Department of Pharmacology, Faculty of Medicine, Chiang Mai University, Chiang Mai 50200, Thailand; warangkana@gmail.com; 3 McCormick Faculty of Nursing, Payap University, Chiang Mai 50000, Thailand; joi.kanjana@gmail.com; 4 Department of Pharmaceutical Sciences, Faculty of Pharmacy, Chiang Mai University, Chiang Mai 50200, Thailand; chsunee@gmail.com; 5 Department of Pharmacognosy, Faculty of Pharmacy, Mahidol University, Bangkok 10400, Thailand; noppamas.sup@mahidol.ac.th; 6 Department of Preclinical Science, Division of Pharmacology, Faculty of Medicine, Rungsit Campus, Thammasat University, Pathum Thani 12120, Thailand

**Keywords:** *Eurycoma longifolia* Jack, extract, anti-inflammatory, antinociceptive effect, antipyretic effects, gastroprotective activity, gastric ulcer, Thai traditional medicine

## Abstract

Tongkat ali (*Eurycoma longifolia* Jack) (ELJ) is a plant in the Simaroubaceae family. Its roots are used in traditional Thai medicine to treat inflammation, pain, and fever; however, the antiulcer abilities of its ethanolic extract have not been studied. This study examined the anti-inflammatory, antinociceptive, antipyretic, and gastroprotective effects of ethanolic ELJ extract in animal models and found that ELJ effectively reduced EPP-induced ear edema in a dose-dependent manner and that a high dose of ELJ inhibited carrageenan-induced hind paw edema formation. In cotton-pellet-induced granuloma formation, a high dose of ELJ suppressed the increases in wet granuloma weight but not dry or transudative weight. In the formalin-induced nociception study, ELJ had a significant dose-dependent inhibitory impact. Additionally, the study found that yeast-induced hyperthermia could be significantly reduced by antipyretic action at the highest dose of ELJ. In all the gastric ulcer models induced by chemical substances or physical activity, ELJ extracts at 150, 300, and 600 mg/kg also effectively prevented gastric ulcer formation. In the pyloric ligation model, however, the effects of ELJ extract on gastric volume, gastric pH, and total acidity were statistically insignificant. These findings support the current widespread use of *Eurycoma longifolia* Jack in traditional medicine, suggest the plant’s medicinal potential for development of phytomedicines with anti-inflammatory, antinociceptive, and antipyretic properties, and support its use in the treatment of gastric ulcers due to its gastroprotective properties.

## 1. Introduction

Inflammation is a complicated response of living tissues to injury, one which can significantly affect health and lifestyle [1]. For decades, inflammation has been treated with anti-inflammatory medications, primarily non-steroidal anti-inflammatory drugs (NSAIDs), although several side effects of those medications have been reported, including nausea, vomiting, dyspepsia, stomach pain, ulcers, and bleeding [2]. One anti-inflammatory drug, cyclooxygenase-2 (COX-2) selective inhibitors, has been linked to cardiovascular side effects [3], prompting the search for suitable alternatives to these often-prescribed drugs in foods and herbs [4,5]. In addition to reducing the risk of adverse effects of long-term exposure to anti-inflammatory drugs, another desirable objective is to identify natural ingredients that not only do not cause gastric ulcers, but which can also reduce their incidence.

*Eurycoma longifolia* Jack (ELJ), also known as tongkat ali, is an herbaceous plant belonging to the Simaroubaceae family which possesses several therapeutic properties. The therapeutic pharmacological effects of ELJ are attributed to the presence of numerous bioactive chemicals, including quassinoids, tirucallanes of the triterpene type, squalene derivatives, eurycomalactone, and bioactive steroids [6]. Various biologically active compounds found in ELJ roots, stems, and leaves, and even in bark, are linked to various pharmacological effects [7]. In the Thai medical tradition, the roots of ELJ have antipyretic properties extracted by boiling 1 handful of dried roots (weighing 8–15 g) with drinking water before breakfast and dinner twice daily [8,9]. In addition, ELJ root extracts have been used as analgesic and anti-inflammatory treatments [10,11]. Furthermore, root preparations of ELJ have been used to treat various illnesses, including malaria, fever, impotence, and loss of sexual desire [12,13,14]. In previous studies, the analgesic activity of ELJ was evaluated using the hot plate and acetic acid tests in mice, and its anti-inflammatory effect was demonstrated in carrageenan-induced paw edema in mice [15]. Notably, it is a component in the Chantaleela recipe, which in Thai traditional folk medicine is prescribed for fever relief and as an anti-inflammatory [14,16]. Apart from antipyretic effects and anti-inflammatory uses, it has been shown that “Radix” herbal remedies made from *Althaea officinalis* L., including ELJ, can successfully protect gastric mucosa against ethanol-induced gastric lesions [17]. Although ELJ roots have been used in Thai traditional medicine, particularly in the Chantaleela formula, which has exhibited efficacy as an antipyretic treatment [16], there have been limited animal investigations on the anti-inflammatory, antipyretic, or gastroprotective effects of ELJ.

To investigate the therapeutic characteristics traditional medicine has attributed to this medicinal plant, we studied the anti-inflammatory and antinociceptive effects of the ethanolic root extract of *Eurycoma longifolia* Jack (ELJ) in vivo on experimental animals. As various non-steroidal anti-inflammatory medicines (NSAIDs) frequently produce mucosal lesions in human stomachs, we also investigated the gastroprotective effect of ELJ extracts using models of gastric ulcers.

## 2. Materials and Methods

### 2.1. Chemicals and Reagents

Aspirin, phenylbutazone, morphine, and prednisolone were purchased from Schering (Bangkok) Ltd., Bangkok, Thailand. Absolute ethanol, carrageenan, ethyl phenylpropiolate (EPP), scopoletin, umbelliferone, cimetidine, and indomethacin were obtained from Sigma Chemical Company (St. Louis, MO, USA). All other chemicals were of analytical grade.

### 2.2. Extract Preparation

*Eurycoma longifolia* Jack (ELJ) was obtained from Vejpong-Osot Drug Store in Bangkok, Thailand. Associate Professor Dr. Noppamas Soonthornchareonnon conducted the species identification, including comparing the ELJ preparation to an authentic sample of the crude drug at the Faculty of Pharmacy Plant Museum, Mahidol University, Bangkok, Thailand, and evaluating its properties in accordance with Thai Herbal Pharmacopoeia methods, e.g., organoleptic examination, extractive values, % loss on drying, total ash, and acid insoluble ash [18]. To prepare an ethanolic extract of ELJ, 5 kg of dried ELJ was macerated in 18 L of 95% ethanol for 5 days, then filtered through Whatman No. 1 filter paper (Sigma-Aldrich, St. Louis, MO, USA) and concentrated at 40 °C using a rotary evaporator (EYELA, Tokyo, Japan). The root extract of *Eurycoma longifolia* Jack yielded a weight-to-weight ratio of 1.80%.

### 2.3. Phytochemical Constituents of E. longifolia as Determined by TLC

The chemical constituents of the ethanolic ELJ extracts were investigated using a modified Tung et al. approach, utilizing thin-layer chromatography (TLC) [19]. The ELJ extract was spread on aluminum plates precoated with silica gel 60 GF (254) (Merck, Darmstadt, Germany), and dichloromethane:methanol (95:5) was used for the mobile phase. The reference standards were scopoletin and eurycomalectone. After development, the TLC plates were placed in a drying chamber. Components were detected under 254 nm and 366 nm UV light, and the plate was then sprayed with 10% KOH, anisaldehyde-sulfuric acid, anisaldehyde-sulfuric acid, and a natural product spraying reagent. The migration distances of unidentified points were compared to standard compounds using Rf values, which were determined as follows: Rf = spot migration distance/solvent migration distance.

### 2.4. Identification of Phytochemicals by High-Pressure Liquid Chromatography (HPLC)

As in previous studies, each sample was analyzed using high-performance liquid chromatography (HPLC) [20,21]. Briefly, a Spheri-5 RP18 column, 220 mm × 4.6 mm i.d. (Perkin-Elmer^®^, Waltham, MA, USA) HPLC column, was used. Mobile phases consisted of methanol and water (content 0.02% phosphoric acid) (23:77) with isocratic systems at a 1.2 mL/min flow rate. A quantum of 20 μL of each sample was injected into the column, with a flow rate of 1.0 mL/min, and monitored with a diode array detector at 345 nm; the column temperature was kept at 23–25 °C.

### 2.5. Animals

Male Sprague-Dawley rats weighing 180 and 200 g, bred by the National Laboratory Animal Center, Mahidol University, Nakhon Pathom, Thailand, were used in this study. Standard animal care was maintained throughout the study period in accordance with the laws and regulations governing animal care and use. The room was kept at 25 ± 1 °C and 60% humidity, with a 12-h day/night interval. Food and water were provided ad libitum throughout the study. After transfer to the animal room, all animals received at least 1 week of care before the experiment began. All animal studies were approved by the Research Ethics Committee for Animal Studies (Study code: 0003/2008), Faculty of Medicine, Thammasat University, Pathum Thani, Thailand.

### 2.6. Test Substance Administration

In each animal experiment, the control group received the same route and volume of vehicles as the test group. In the ear edema model, 20 µL/ear of ELJ extracts and a reference drug (ibuprofen) were applied topically to the ears of rats. A total of 5 mL/kg body weight of ELJ extracts and reference drugs (aspirin, morphine, and prednisolone) were administered by oral gavage to the other models.

### 2.7. Anti-Inflammatory Activity

#### 2.7.1. Ethyl Phenylpropiolate (EPP)-Induced Ear Edema

To investigate the topical anti-inflammatory impact of ELJ extracts, a modified version of the method reported by Brattsand et al. (1982) [22] was used. Thirty male Sprague-Dawley rats weighing 40–60 g were randomly assigned into five groups of six rats each. EPP was dissolved in acetone and topically administered to the inner and outer surfaces of both ears at a dose of 1 mg/ear. The acetone (vehicle control), ELJ extracts (1, 2, and 4 mg/ear), or ibuprofen (1 mg/ear) were applied to the ear immediately before EPP administration. The ear thickness was measured with digital vernier calipers beforehand, and at 15, 30, 60, and 120 min after edema induction. The test substances’ ear edema inhibition was then determined. The percentage of inhibition was calculated by comparing the increase in ear thickness of each test group to that of its control group.

#### 2.7.2. Carrageenan-Induced Hind Paw Edema in Rats

Thirty-six rats weighing 100–120 g were divided randomly into six groups (6 rats per group). On the plantar side of the right hind paw of the rats, carrageenan (0.05 mL, 1% *w*/*v* in NSS) was injected intradermally. Aspirin (300 mg/kg, p.o.) or ELJ extracts (300, 600, and 1200 mg/kg) were administered 1 h before carrageenan administration. The edema of the right hind paw was measured beforehand, and at 1, 3, and 5 h after carrageenan injection using a plethysmometer (model 7150, Ugo Basile, Italy) [23]. The degree of decrease in the difference in paw edema volume between the left (without carrageenan) and right (with carrageenan) hind paws were used to determine the anti-inflammatory activity of the ELJ extracts (mL). The percentage of inhibition was estimated by comparing each test group’s decreased paw thickness to that of the corresponding control group.

#### 2.7.3. Cotton-pellet-induced Granuloma Formation in Rats

Twenty-four rats weighing 200 to 250 g were divided randomly into four groups of six each. Two sterile cotton pellets (19–21 mg) were implanted subcutaneously, one on each side of the rat’s abdomen, while the rats were under anesthesia. Each group of rats then received distilled water (0.5 mL/100 g, p.o.), aspirin (300 mg/kg/day, p.o.), prednisolone (5 mg/kg/day, p.o.), or ELJ extract (1200 mg/kg/day, p.o.) daily for 7 days. On the eighth day, the rats were anesthetized, and the implanted pellets were dissected. The pellets’ wet and dry weights (dried at 60 °C for 18 h) were determined. The granuloma inhibition and transudative weight were calculated. The thymus gland dry weight and the animals’ body weight were also measured [24].

### 2.8. Formalin-Induced Nociception Model

The formalin test was conducted using Swiss albino mice following the procedures described above [25]. The behavior of the mice after receiving an intraplanar injection of 20 mL 2.7% formalin (1% formaldehyde) in saline into the ventral surface of the right hind paw was observed. The licking reaction time during the first five minutes following the injection was recorded as the neurogenic or initial response; the licking reaction during the period 20–30 min after injection was recorded as the late or inflammatory pain response. One hour before the formalin injection, the mice were administered either ELJ extracts (300, 600, or 1200 mg/kg) or saline solution (10 mL/kg) orally (by gavage). As an indicator of nociception, the duration of the animals’ licking of the injected paw was measured with a chronometer after formalin injection and placement in 20-cm-diameter glass cylinders. The data were analyzed using the following equation to determine the mean percent inhibition of licking response (PIL).

$$PIL=\frac{Licking\;response\;time\;\left(control\right)-Licking\;response\;time\;(treated)}{Licking\;response\;time\;(control)}\times 100$$

### 2.9. Antipyretic Activity

Thirty male rats weighing 180–200 g, randomly divided into five groups of six rats each, were subjected to the yeast-induced hyperthermia model, following Mizui et al. [26]. A 12-channel electronic thermometer (LETICA, model TMP 812 RS, Panlab S.L., Cornellà de Llobregat, Spain) was used to determine rectal temperatures at baseline. To induce hyperthermia, yeast was injected subcutaneously (1 mL/100 g body weight, 25% brewers’ yeast *w*/*v* in NSS). After 18 h, rectal temperatures were measured again. Dosages of 5% Tween 80, aspirin (300 mg/kg), or ELJ extracts (75, 150, or 300 mg/kg) were administered orally to rats with a temperature increase of greater than 1 °C. Rectal temperatures were measured at 30, 60, 90, 120, and 180 min after treatment, and again after 18 h.

### 2.10. Gastric Ulcer Models

All gastric ulcer experiments were performed in 12 h fasted rats. Water ad libitum was allowed up to 1 h before drug administration. For each experiment, 30 rats were randomly allocated into 5 groups of 6 rats each as follows: group 1 received sterile water (control), group 2 received standard treatment (100 mg/kg of cimetidine), group 3 received 150 mg/kg of ELJ extract, group 4 received 300 mg/kg of ELJ extract, and group 5 received 600 mg/kg of ELJ extract. The rats were monitored for symptoms and any adverse effects. The rats were also given a high dose of ELJ extract (600 mg/kg) daily for 14 days to investigate whether ELJ could cause a gastric ulcer.

#### 2.10.1. Ethanol/Hydrochloric Acid (EtOH/HCl)-Induced Gastric Lesions

The gastric ulceration ethanol evaluation was conducted using an acidified solution, as modified from a published methodology [27], using thirty rats (6 rats per group). One hour after oral administration of the test drugs, 0.1 mL of EtOH/HCl solution (consisting of 60 mL of absolute ethanol, plus 12.5 mL of HCl and 27.5 mL of water) was orally administered to the rats in all groups. After one hour, the rats were sacrificed, using thiopental sodium induction, before evaluating gastric lesions. An incision was made along the greater curvature of the stomach, exposing the gastric mucosa. The size of the gastric ulcers was measured in millimeters (mm) under a 10-x microscope. The size of the lesions was converted to an ulcer index (UI), using the following formula:

(1) $$UlcerIndex(UI)=\frac{Sum\;of\;the\;total\;length\;of\;lesions\;in\;each\;group}{Number\;of\;rats\;in\;that\;group}$$

Then, the percentage of gastric ulcer inhibition (% inhibition) of each test drug was estimated using the following formula:

(2) $$\%\mathrm{Inhibition}=\frac{UIc-UIt}{UIc}\times 100$$

where UIc is the “ulcer index of the control group” and UIt is the “ulcer index of the test group”.

#### 2.10.2. Indomethacin-Induced Gastric Lesions

Indomethacin is known to cause significant adverse reactions, including petechial hemorrhage, inflammatory lesions, and erosions of the stomach mucosa [28,29,30]. A dose of 30 mg/kg indomethacin suspension in 5% Tween 80 was administered intraperitoneally to each of the animals in all groups. Five hours later, the rats were sacrificed to determine gastric lesions. Ulcer indexes and % inhibition of gastric ulcers were calculated.

#### 2.10.3. Restraint Water Immersion Stress-Induced Gastric Lesions

Water immersion and stress-induced gastric lesions in rats, a combination of physical and psychological stressors, resulted in lesions that mimicked those caused by sepsis, trauma, or surgery [31,32]. After the rats had been fasted for 48 h without food and 1 h without water, they were randomly divided into 5 groups of six each, and the test drugs were administered orally. One hour after drug administration, the rats were placed in stainless steel cages that fit only one animal per cage, with the head upright for 5 h, and the cages immersed in a cold-water tank (20 ± 2 °C). The water level was maintained at the rats’ chest level. The rats were then sacrificed, and the gastric lesions were measured. The ulcer indexes and % inhibition were compared between groups.

### 2.11. Pylorus Ligation

One hour after drug administration, general anesthesia was administered to each rat. Laparotomy and pyloric ligation were performed, followed by skin closure [33]. Five hours later, the animals were sacrificed. Their stomach and gastric contents were removed. After being centrifuged at 2500 rpm for 5 min, the gastric juice was tested for total volume and pH, as well as total acidity in the supernatant. Quantifying the total acidity of gastric juice was performed by titration with 0.1 N NaOH to an endpoint of pH 7.4 using phenolphthalein as the indicator to determine the amount in ml and µEq per 100 g body weight of the rat each hour. Gastric ulcers were measured at their greatest length (mm), and then UI and percentage of ulcer inhibition were calculated.

### 2.12. Statistical Analysis

One-way analysis of variance (ANOVA) and the post hoc least significant difference (LSD) test were used to compare the data between groups using GraphPad Prism 9.0 software (GraphPad Software, Inc., San Diego, CA, USA). Data are shown as mean ± standard error of the mean (SEM). *p*-values less than 0.05 were considered statistically significant.

## 3. Results

### 3.1. Specification of E. longifolia Jack by TLC and HPLC Determinations

Screening for phytochemicals was performed on the root ELJ in accordance with the protocol for the standard method. The saponin, the terpenoids, and an unidentified blue-colored compound were found in the tested sample. The results of their respective quality tests, which were conducted according to the 2018 Thai Herbal Pharmacopoeia, are shown in Table 1.

**Figure 1:**
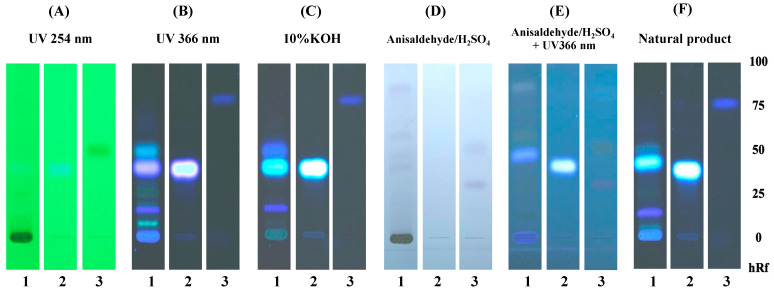
TLC chromatogram of 95% ethanolic extract of *Eurycoma longifolia* Jack: TLC chromatogram of ethanolic extract of *Eurycoma longifolia* Jack (1), scopoletin (2), and eurycomalectone (3), observed under (**A**) UV at 254 nm, (**B**) UV at 366 nm, (**C**) 10%KOH, (**D**) anisaldehyde–sulfuric-acid reagent, (**E**) anisaldehyde–sulfuric-acid reagent with UV at 366 nm, and (**F**) natural product spraying reagent.

Scopoletin and eurycomalectone were selected as reference standards, and their Rf values were measured, as represented in Figure 1. Scopoletin was seen as spots under 254 and 366 nm UV light in 10%KOH, an anisaldehyde–sulfuric-acid reagent with UV at 366 nm, and a natural product spraying reagent with Rf = 0.40. While under 254 nm UV light and with an anisaldehyde–sulfuric-acid reagent, eurycomalectone appeared as a spot with Rf = 0.50. The ethanolic extract of ELJ resulted in a blue band with Rf = 0.40 under 254 and 366 nm UV light, 10%KOH, an anisaldehyde–sulfuric-acid reagent with UV at 366 nm, and natural product spraying, suggesting the presence of scopoletin. Unlike the standard eurycomalectone, the ethanolic extract ELJ exhibited bands under 366 nm UV light, 10% KOH, and a natural product spraying reagent with Rf = 0.52. Consistent with the previous study, it was reported that scopoletin and eurycomalectone are components of an ethanolic extract of *E. longifolia* Jack [34,35].

**Figure 2:**
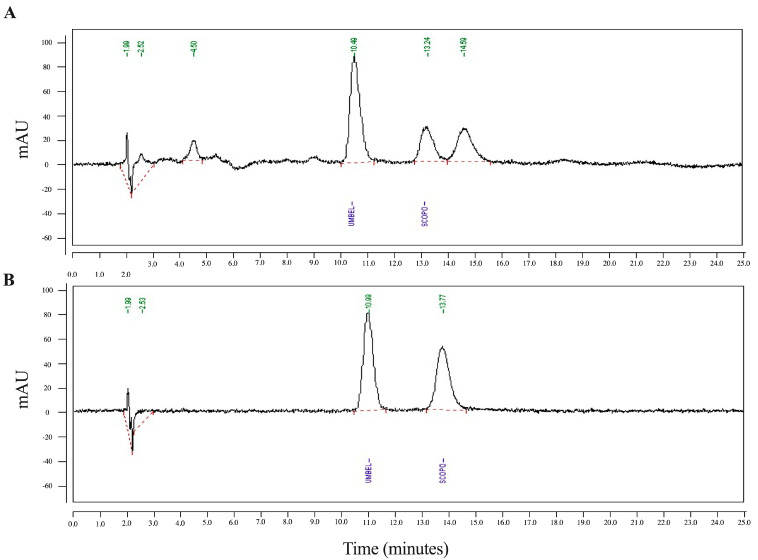
The HPLC of scopoletin of ethanolic ELJ extract (**A**) compared to standard (**B**). Umbelliferone used as an internal standard, with a retention time well-separated from the scopoletin, was mixed with scopoletin to correct for volume errors.

Apart from TLC, scopoletin was also seen as a clear peak in HPLC, and the amount of it in the ELJ extract was then quantitatively compared to the standard (Figure 2A) and umbelliferone with a well-separated retention time from scopoletin was used as an internal standard to correct for volume errors (Figure 2B). According to HPLC analysis, scopoletin accounted for 12.590 µg/mL of the ELJ extract, which was determined to be 0.252% *w*/*w* of crude extract. The result implies that scopoletin in TLC results were consistent with HPLC analyses. Therefore, the presence of scopoletin and eurycomalectone in our study on the analyzed ELJ extracts was consistent with the previous research. In the future, the TLC technology could be paired with mass spectrometry for the identification of chemical components that have been separated.

### 3.2. EPP-Induced Ear Edema

**Figure 3:**
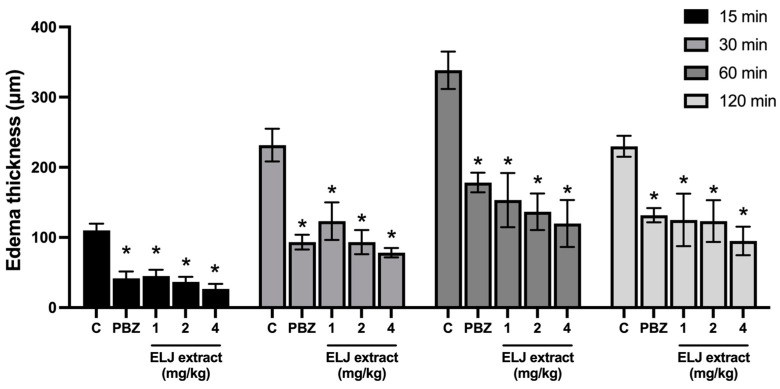
Effects of ELJ extract and phenylbutazone on EPP-induced ear edema in rats. Values are expressed as mean ± SEM (*n* = 6). Control: treated with acetone. * Significantly different from its control group, *p* > 0.05. C, Control. PBZ, phenylbutazone (1 mg/kg).

As indicated in Figure 3, EPP applied topically to the ears of rats caused ear edema after 15 min. The maximal effect lasted one hour and then progressively diminished. All treatments, including ELJ extracts and phenylbutazone, significantly decreased ear edema formation with equivalent inhibition percentages at all evaluation time points.

### 3.3. Carrageenan-Induced Hind Paw Edema

**Figure 4:**
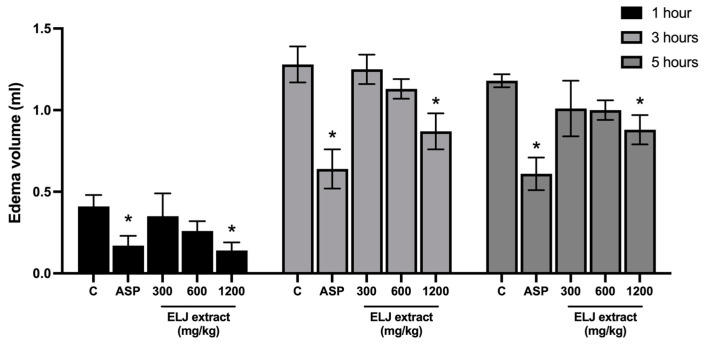
Effects of ELJ extracts and aspirin on carrageenan-induced hind paw edema in rats. Values are expressed as mean ± SEM (*n* = 6). Control: treated with acetone. * Significantly different from its control group, *p* > 0.05. C, Control. ASP, Aspirin (300 mg/kg).

Figure 4 shows the inhibitory effect of oral treatment of ELJ extracts on carrageenan-induced paw edema in rats. The injection of carrageenan caused edema of the hind paw within 1 h, with the maximum effect occurring after 3 h. Only ELJ extract at a high dose (1200 mg/kg) and aspirin (300 mg/kg) effectively prevented hind paw edema formation at all assessment time points, with the greatest inhibition at 1 h after carrageenan administration. This inhibitory impact of ELJ extracts appeared to be dose dependent.

### 3.4. Cotton-Pellet-Induced Granuloma Formation

Aspirin (300 mg/kg/day) and ELJ extract (1200 mg/kg/day) tended to reduce transudative weights and granuloma weights, as shown by their granuloma inhibition of 4% and 25%, respectively, except for prednisolone (5 mg/kg/day), which significantly reduced from those of control groups, as shown by their granuloma inhibition of 37%. (Table 2). As shown in Table 3, the body weight gain and dry thymus weight did not differ substantially between the prednisolone, aspirin, and ELJ extract groups.

### 3.5. Formalin-Induced Nociception Model

In the formalin test, the control group’s average licking duration was 67.8 s in the early phase and increased to 93.8 s in the late phase (Table 4). All treatments (aspirin, morphine, and ELJ extracts) significantly inhibited the paw licking in both phases in a dose-dependent manner. In the early phase, the percentage inhibition of paw licking by ELJ extracts at 300, 600, and 1200 mg/kg were almost the same, accounting for 34%, 38%, and 43%, respectively. However, in the late phase, maximum inhibition was recorded for aspirin (100%), followed by morphine (98%) and ELJ extracts ranging from 88–97%, which was comparable to standard.

### 3.6. Antipyretic Activity

As shown in Table 5, the rectal temperature of all mice was increased 18 h after the baker’s yeast injection. Aspirin at 300 mg/kg and ELJ extract at 1200 mg/kg dose significantly reduced hyperthermia at all time points (Table 5).

### 3.7. Anti-Ulcerogenic Activities of ELJ Extract in Gastric Ulcer Models

**Figure 5:**
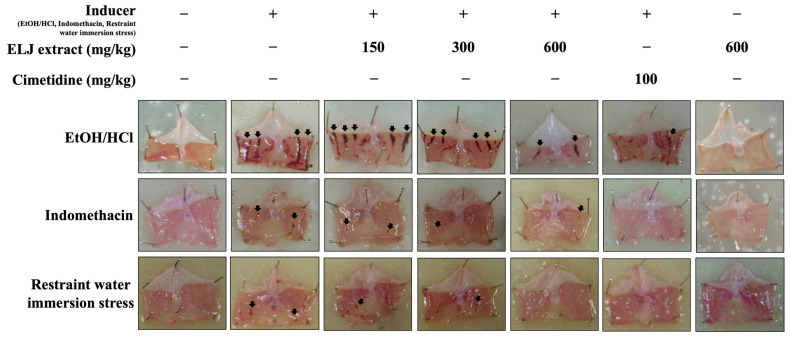
Evaluation of the effect of *Eurycoma longifolia* Jack (ELJ) extract at 150, 300, and 600 mg/kg and cimetidine at 100 mg/kg on the gastric surface in rats, comparing three different models of gastric ulcers to the control group. The black arrows represent the typical necrotic bands or spots and small erosions that can develop into gastric ulcers.

Administration of ELJ to rats at the highest dose (600 mg/kg) for 14 days did not result in the development of stomach ulcers (Figure 5). The anti-ulcerogenic effect of ELJ extract was investigated using three gastric ulcer models. The EtOH/HCl-acid-induced gastric lesions model results in the most severe gastric mucosal injury in several hemorrhagic areas in the glandular part of the stomach. Administration of both ELJ extracts at 150, 300, and 600 mg/kg and cimetidine at 100 mg/kg appeared to reduce stomach lesions compared to the induction group. In comparison to the EtOH/HCl-acid-induced gastric lesions model, the indomethacin and restraint water immersion stress models produced fewer spots and less damage to the stomach glandular mucosa. In both models, stomach mucosal damage was reduced after treatment with either ELJ extract or cimetidine. In addition, there was no evidence of harm to the stomach in the group that received only ELJ extract.

#### 3.7.1. Effect of ELJ on the EtOH/HCl-Induced Gastric Ulcer in Rats

**Figure 6:**
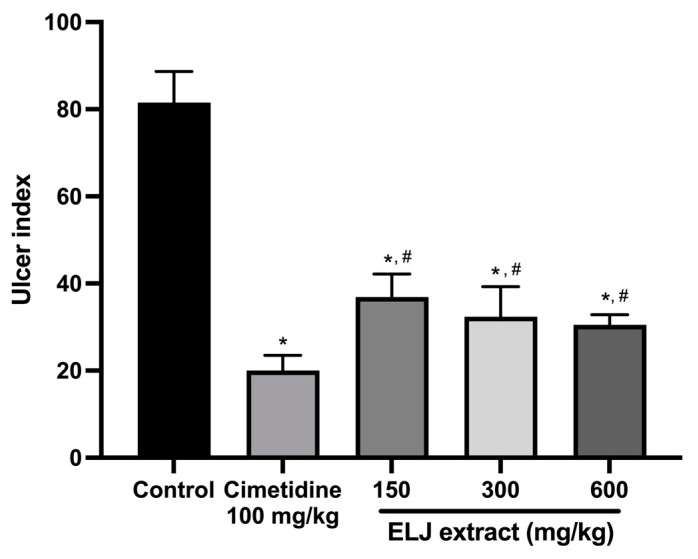
Effects of ELJ extract, control, and cimetidine in a rat model of EtOH/HCl-acid-induced gastric lesions. Ulcer indexes are mean ± S.E.M. (*n* = 6). * Significantly different from the control group, *p* < 0.05; # Significantly different from the cimetidine group, *p* < 0.05.

In the EtOH/HCl-acid-induced gastric lesions model, ELJ extract at 150, 300, and 600 mg/kg demonstrated gastric ulcer inhibition levels of 55, 60, and 62%, respectively (Supplementary Table S3). The ulcer indexes were dramatically reduced compared to the control group after treatment with 150, 300, and 600 mg/kg of ELJ extract (Figure 6). Furthermore, ELJ extract had identical ulcer indexes and inhibition percentages at higher doses of 300 and 600 mg/kg. Additionally, cimetidine was found to have a more pronounced effect on reducing gastric ulcers than did ELJ extract, suggesting that treatments with ELJ extract may help reduce and prevent gastric lesions induced by EtOH/HCl.

#### 3.7.2. Effect of ELJ and Cimetidine on Indomethacin-Induced Gastric Ulcer in Rats

**Figure 7:**
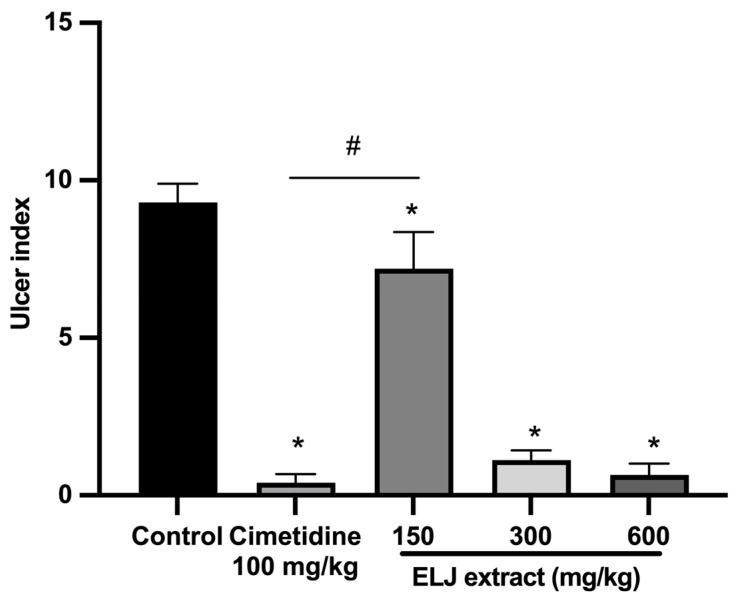
Effect of ELJ and cimetidine on indomethacin-induced gastric ulcers in rats. Ulcer indexes are mean ± S.E.M. (*n* = 6). * Significantly different from the control group, *p* < 0.05; # Significantly different from the cimetidine group, *p* < 0.05.

With an ulcer index of 9.30 ± 0.59 mm, the indomethacin-induced damage to the gastric glandular mucosa of the control group was evident (Supplementary Table S4). To the contrary, at all doses, rats treated with either cimetidine or ELJ exhibited dramatically reduced gastric lesions. At a dose of 600 mg/kg, ELJ inhibited gastric ulcers by a maximum of 93%, whereas cimetidine inhibited ulcers by 95% (Figure 7).

#### 3.7.3. Effect of ELJ on Restrained Water Immersion Stress-Induced Gastric Ulcer in Rats

**Figure 8:**
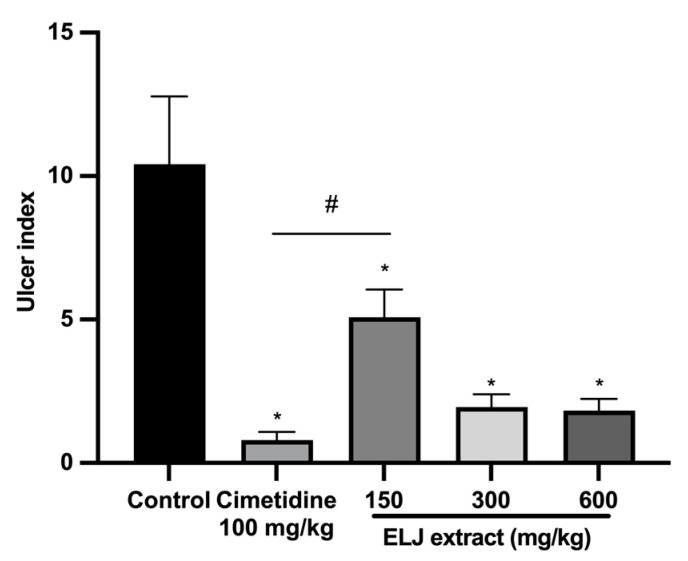
Effects of ELJ extract, control, and cimetidine in a rat model of the restrained water immersion stress-induced gastric lesions. Ulcer indexes are mean ± S.E.M. (*n* = 6). * Significantly different from the control group, *p* < 0.05; # Significantly different from the cimetidine group, *p* < 0.05.

ELJ extract at doses of 150, 300, and 600 mg/kg and cimetidine at a dose of 100 mg/kg significantly reduced gastric ulcer formation induced by restrained water immersion stress in the rats (Figure 8). The percentage of inhibition from cimetidine at a dose of 100 mg/kg and ELJ extract at doses of 150, 300, and 600 mg/kg were 95, 71, 89, and 89% in the restrained water immersion stress-induced gastric lesions model (Supplementary Table S5). These findings suggest that ELJ extract might have the ability to reduce the severity of chemically and physiologically induced gastric ulcers.

### 3.8. Pylorus Ligation Model

None of the doses of ELJ extract reduced the gastric acid’s secretory rate or total acidity, or increased the intragastric pH in the pylorus ligation rat model (Table 6). Conversely, the secretory rate and total acidity in rats receiving 100 mg/kg of cimetidine decreased significantly compared to the controls.

## 4. Discussion

Presently, medical herbs are commonly used in the context of food additives for health maintenance, and plant products have also been increasingly found in the market, because herbs contain several natural materials, including phenolic acids and flavonoids, that are used in the treatment of diseases [36,37]. *E. longifolia* leaf decoctions are used to wash away itches, while its root bark is used to treat gastric ulcers, diarrhea, and fever. Additionally, its roots are utilized as an appetite stimulant and nutritional supplement [38]. Numerous investigations on the bioactivity of this plant have been conducted; however, the anti-inflammatory, antinociceptive, and antipyretic activities of ethanolic *E. longifolia* Jack (ELJ) and its protective efficacy against gastric ulcers have not been investigated. The present study focuses on ELJ’s antiulcer potential in animal models.

Apart from extraction, ethanol is commonly used to screen for phytoconstituents in plant extracts because of the ease with which it penetrates the cellular membrane, thus gaining access to intracellular targets [39]. Additionally, plants used in ethanolic extraction contain phytochemicals such as phenolic acids and flavonoids, which may be directly comparable to other solvents (e.g., hexane, ethyl acetate, or acetone), and ethanol can be used effectively to treat industrial waste [40]. Extraction methods increase composite amounts depending on the solvent used and the extraction process [41]. In 2010, Huang and Chang reported that ethanol may offer a higher yield [42]; in this study, we therefore chose to use ethanol for extraction.

From the chemical pattern of the effect of ELJ extract on TLC and HPLC, which was found in this study, we now qualify the efficacy of herbs using a control model approach following the Thai Herbal Pharmacopoeia (THP) formula [18] to generate the monograph of this plant. TLC studies have demonstrated that our ELJ extract contains scopoletin, while phytochemical screening revealed the presence of phenolics, tannins, flavonoids, and terpenes [43]. In addition to promoting wound healing, these compounds have been demonstrated as displaying antioxidant properties. In fact, the antioxidant activity of ELJ extract is positively correlated with flavonoid contents [44]. TLC fingerprints of the ELJ extract (Figure 1) were compared with the reference markers, such as scopoletin. Compared to eurycomalectone, the chromatographic pattern of the ELJ extract was noticeably weaker. Thus, eurycomalectone in ELJ extract contained fewer chemical elements than did the other samples, indicating that it was less effective. This discrepancy could have been caused by differences in the environmental conditions under which the plants were grown, or by the age of the plants when they were picked, among other factors [45]. The scopoletin TLC pattern resembles the ELJ extract pattern which we have observed in this study (Figure 1). Along with the first two components of ELJ (scopoletin and eurycomalectone), quassinoids are another crucial component that has been shown to protect against gastric ulcers in rats with pylorus ligation and indomethacin-induced gastric lesions [46]. The fractionation approach should be investigated further to study phytochemicals and active substances. In addition, other quassinoids, such as eurycolactone A, B, D, E, and F, process anti-inflammatory properties, while both eurycolactone and eurycomanol regulate signaling pathways involved in cell development, cell death, and inflammation [47,48].

In a previous study of an aqueous extract of *Eurycoma longifolia* Jack (ELJ) on reproductive functions in the rat [49], the effective dose range for ELJ was 200–800 mg/kg of body weight. This range of ELJ extracted by methanol or water has been used in various investigations [50,51]. The gastroprotective efficacy of ELJ extract administered at a dose of 100 mg/kg, compared to that of the standard drug cimetidine (100 mg/kg), for the prevention of development of acute gastric ulcers was investigated in restrained rats [52]. Based on that study, a dose range of 150–600 mg/kg body weight was selected as a safe and efficacious concentration for the ELJ administration to rats in this study. Due to its safety, the European Food Safety Authority (EFSA) classifies ELJ as a new food in Europe, and a 12-month chronic toxicity effect in rats at 250, 500, and 1000 mg/kg bodyweight per day revealed no toxicity for ELJ extract [53]. Moreover, Thailand Traditional Medicine has used it for years to treat malaria [14,16]. These results indicate that ELJ extract is safe for both animals and humans. Using various animal models, this study could demonstrate, for the first time, the anti-antinociceptive of *Eurycoma longifolia* Jack, but not the anti-inflammatory and analgesic activities.

ELJ has been a procedure widely used to screen and assess the anti-inflammatory activity of test compounds using the EPP-induced ear edema mode [22]. EPP triggers the release of pro-inflammatory mediators, including histamine, serotonin, kinins, and prostaglandins, resulting in an acute inflammatory response that causes vascular alterations, including vasodilation and an increase in vascular permeability, resulting in the creation of ear edema [54,55]. This study used phenylbutazone as a reference drug to inhibit cyclooxygenase (COX) [56]. Those pro-inflammatory mediators, as well as both phenylbutazone and ELJ extracts, effectively reduced EPP-induced ear edema. This indicates that the potential mechanism of action of ELJ extracts may involve the suppression of the production of these pro-inflammatory mediators.

Injecting carrageenan into the plantar surface of the hind paw induces an initial inflammatory reaction that results in a biphasic phase of paw edema, and it is a well-established model for testing the anti-inflammatory efficacy of various treatments [57,58]. During the first phase following carrageenan injection (0–2.5 h), kinins, serotonin, and histamine are released. The second phase (2.5–6 h) is characterized by the release of bradykinin, NO, and PGs, which are associated with the elevation of COX-2 and nitric oxide synthase (iNOS) activities, as well as oxygen-derived free radicals, in addition to neutrophil infiltration and activation in the injured areas [58,59]. For our current study, only an oral treatment with a high dose of either ELJ (1200 mg/kg) or aspirin (300 mg/kg) significantly reduced the production of carrageenan-induced rat paw edema during all measurement periods, demonstrating the recipe’s ability to modify the intensity of inflammation.

Cotton-pellet-induced granuloma formation, whose responses can be classified into three stages, is commonly used to examine the anti-inflammatory effects of test compounds on chronic inflammation [57]. The initial or transudative phase, which occurs 0–3 h following cotton pellet implantation, is characterized by fluid leaking from blood vessels due to increased vascular permeability. The second or exudative phase occurs 3–72 h after cotton pellet implantation, and is characterized by protein leaking from the bloodstream around the granuloma due to the significant increase in vascular permeability. The final or proliferative phase, lasting 3–6 days, is characterized by the development of granulomatous tissues resulting from the continual release of pro-inflammatory mediators [24,60]. According to Swingle and Shideman (1972), the weight of the fluid in the granuloma is correlated with the transudative component. In contrast, the weight of the dry pellet is associated with the amount of granuloma tissue deposited. In terms of therapy, steroids can decrease both the transudative and proliferative stages significantly, whereas NSAIDs, such as aspirin, can reduce these effects only moderately [61]. The present study showed that, although ELJ extract was unable to reduce transudative weight or suppress granuloma formation effectively, these effects were more pronounced when compared to aspirin but less pronounced when compared to prednisolone. Furthermore, the thymus’ weight and overall weight gain were unaffected by the ELJ extract. Thus, it appears improbable that ELJ extract has a steroid-like action. Taken together, these data suggest that ELJ extract can reduce chronic inflammation with an effect superior to that of aspirin but inferior to that of prednisolone. However, the mechanical effects of the ELJ extract need to be further explored.

The formalin test is a suitable and exhaustive model for testing the antinociceptive efficacy of pharmaceuticals. The early neurogenic pain phase is caused by the direct stimulation of nociceptors, whereas the late inflammatory pain phase is caused by the release of pro-inflammatory mediators [62]. In this investigation, all ELJ extract treatments significantly inhibited paw licking responses in both phases (Table 4). This finding indicated that ELJ extracts have analgesic efficacy through reducing early neurogenic pain and, in particular, late inflammatory pain. The percentage inhibition of licking response in the late phase was higher than in the early phase for all treatments, with comparable effects to aspirin, possibly due to decreased production or preferred inhibition of pro-inflammatory mediators such as prostaglandin, adenosine, and histamine in peripheral tissue.

It is generally believed that yeast-induced hyperthermia is caused by the activation of endogenous pyrogen and the consequent synthesis of pro-inflammatory mediators, which then act on the hypothalamus and drive prostaglandin E2 (PGE2) synthesis in the preoptic area of the anterior hypothalamus thermoregulatory centers [63,64]. The inhibition of prostaglandin synthesis could be a mechanism of antipyretic action comparable to that of acetaminophen and aspirin [65], and the inhibition of prostaglandin can be achieved by decreasing the activity of the cyclooxygenase enzyme. Several mediators contribute to fever, and their suppression is responsible for the antipyretic effect [66]. Only a high dose of ELJ extract (1200 mg/kg) effectively reduced the rectal temperature of yeast-infected rats. Thus, it can be hypothesized that the ELJ extract contained pharmacologically active components that could inhibit prostaglandin release. This result led to the hypothesis that ELJ extract could exert central antipyretic effects.

Since the undesirable gastrointestinal side effects of existing anti-inflammatory medications are major downsides, patients and physicians will welcome new anti-inflammatory drugs that could help avoid these problems, such as ELJ, an extract derived from a flowering plant *Eurycoma longifolia* Jack (*Tongkat ali*) of the family Simaroubaceae, which is native to Indonesia, Myanmar, Laos, and Thailand [6]. Most NSAIDs used to treat inflammation and pain also result in stomach ulcers [2]. We administered ELJ extract to rats for fourteen days, after first investigating its impact on gastric ulcers in rats. It was found that EJL extract could not be the cause of gastric ulcers. Subsequently, rats administered 150 to 600 mg/kg of ELJ extract were examined to determine whether it could prevent gastrointestinal tract ulcers.

Acidified ethanol can be administered orally, and is known to cause intracellular oxidative stress in the gastric mucosa, as well as disrupt gastric mucus, which can perturb superficial epithelial mucosa, cause cellular necrosis, and cause a gastric ulcer [67]. Moreover, the EtOH/HCl model showed that hydrochloric acid induces gastric ulcer formation via direct irritation of the gastric mucosa, while ethanol decreases gastric mucous production, endogenous glutathione, mucosal blood flow, and prostaglandins [68,69]. The results of this study show that rats given ELJ extract at doses of 150, 300, and 600 mg/kg had considerably lower ulcer indexes than did the controls. According to previous research, an aqueous extract of *E. longifolia* includes significant amounts of superoxide dismutase (SOD) and hydroxyl radical scavenging capabilities. SOD is an antioxidant that plays a crucial role in preventing oxidative stress. Thus, it is probable that the antioxidative impact of ELJ extract contributes to the reduction of acidified-alcohol-induced gastric ulcer formation. These findings are comparable with studies of the cimetidine group, indicating that ELJ extract exhibits gastroprotective activity. However, the mechanism of ELJ extract’s antioxidant activity in relation to the EtOH/HCl-induced gastric ulcer model should be explored for further.

The use of NSAIDs such as indomethacin typically causes gastric ulcers by disrupting the natural gastric mucosal barrier of hydrophobic mucous and bicarbonates. Inhibition of the cyclooxygenase-1 (COX-1) enzyme disturbs cytoprotective prostaglandin synthesis [70]. Additionally, indomethacin can cause direct damage to the mucosa by uncoupling the mitochondrial oxidative phosphorylation, which leads to an increase in the formation of reactive oxygen species (ROS) [71]. The anti-ulcerogenic effects were confirmed in the indomethacin-induced gastric lesion model. At different dosages of ELJ extract, the ulcer indexes were statistically significantly lower than those of the controls. At a high dose of ELJ extract (600 mg/kg), the ulcer index of treated rats was non-inferior to the 100 mg/kg group of cimetidine-treated rats.

When experimental rats are exposed to cold water, stress can induce vagal overactivity, thereby increasing gastric acid secretion, while decreasing gastric mucosal blood flow, leading to gastric ulcer formation [72]. As demonstrated in the restrained water immersion stress-induced gastric lesion model, the ulcer indexes of the ELJ-treated groups were significantly lower than that of the non-ELJ-treated group and were almost the same as that of the cimetidine-treated group.

In addition to the EtOH/HCl and indomethacin-induced peptic ulcer models, macroscopic and microscopic characteristics are critical for elucidating cellular mechanisms. Both models give total scores generated from similar partial evaluation scores at the macroscopic level (size, number, and site of hemorrhagic lesions). When anti-ulcerative treatments were studied macroscopically, gastric lesions were decreased, most notably with cimetidine and ELJ extract at a high doses (600 mg/kg). In both animal models, anti-ulcerative medicines significantly reduced the depth of mucosal erosion [73,74]. However, the microscopic evaluation score, which includes erosion depth, the severity of hemorrhage, inflammation, and apoptosis, should be investigated further to identify the possible mechanisms of action of the ELJ extract.

Pyloric ligation is a surgical model used to determine anti-secretory activity. Ligating the pylorus causes intraluminal HCl accumulation, which aggressively damages the gastric mucosa [75]. In this study, gastric acid output was examined in all groups, but only the animals in the cimetidine group showed a significant reduction of gastric acid secretory rates and total acidity. ELJ extract did not decrease the volume of gastric acid or increase the intragastric pH. Scopoletin, a purified compound found in ELJ extract, has been shown to have a preventative effect against the development of esophagitis [76]. In the present study, however, it is probable that the concentration of scopoletin in ELJ extract at 12.590 g/mL was insufficient to suppress gastric acid secretion and total acidity (Table 6). Moreover, obtaining enough scopoletin to use in each experiment was problematical, because the amount of ELJ extract available was limited. Cimetidine is an antagonist of histamine-2 receptors (HH2R) that controls gastric acid secretion by parietal cells. Thus, the results indicate that the anti-ulcerogenic activity of ELJ extract is not related to its anti-secretory activity.

A previous study of a product containing ELJ as a primary constituent on gastroprotective effects and gastrointestinal antioxidants discovered that this product could prevent gastric ulcers in the EtOH-induced gastric lesion model, but could not reduce MDA (malondialdehyde) levels or gastric acid [17]. As previous research on ELJ has been inconclusive, additional study of its protective efficacy against gastric ulcers is needed. The present study investigated the gastroprotective effects of ELJ using three types of gastric ulcers induced in rats by means of acidified ethanol, indomethacin, and restraint water immersion, respectively. In all gastric ulcer models, ELJ extract at concentrations of 150, 300, and 600 mg/kg effectively prevented gastric ulcer formation. The efficacy of ELJ extract at 600 mg/kg was roughly comparable to that of cimetidine at 100 mg/kg, accounting for 62%, 93%, and 82% in the EtOH/HCl-induced, indomethacin-induced, and restrained water immersion stress-induced gastric lesions models, respectively. In contrast, the efficacy in the cimetidine-treated groups was superior to that of ELJ at 75%, 95%, and 92%. In the pyloric ligation model, however, the effects of ELJ extract on gastric volume, gastric pH, and total acidity were not statistically significant, indicating that ELJ extract has potent anti-gastric properties, but has no effect on gastric pH or acidity.

This non-clinical investigation was conducted to examine the gastroprotective effects (i.e., inflammation, fever, and pain) of ELJ extract in vivo using animal models. Some clinical studies have shown that ELJ extract is safe to use, and may even be effective in the induction of immunomodulation by ingestion of capsules containing 200 mg of a standardized *E. longifolia* water-soluble TA (Tongkat Ali) root extract, without inducing toxicity in blood parameters or biochemical analyses [77]. Even though there have been only a few clinical studies on ELJ extract, it is anticipated that the results of this study could lead to future clinical trials to investigate the action of ELJ extract and establish possible mechanisms for its gastric-ulcer protection ability.

## 5. Conclusions

The present study demonstrates that ethanolic extract of *Eurycoma longifolia* Jack extract (ELJ) exerts dose-dependent anti-inflammatory and antinociceptive action against formalin-induced nociception, whereas only a high dose of ELJ extract has antipyretic activity. This study provides scientific evidence, in the preclinical phase, of the pharmacological activity of *E. longifolia* extract in its anti-inflammatory, antinociceptive, antipyretic, and gastroprotective activities and supports its use, without causing gastric ulcers, in gastric ulcer treatment. For these reasons, ELJ extract should be regarded as having advantages over some NSAIDS. Prior to promotion of routine clinical use, additional preclinical studies of its attributes, e.g., short- and long-term pharmacological activities and toxicity, are needed to evaluate the safety ELJ extract.

## Figures and Tables

**Table 1:** Physical and chemical properties of the root of *E. longifolia* Jack.

Test	Result
Foreign matter (%*w*/*w*)	Not found
Hexane extractive (%*w*/*w*)	0.66
Dichloromethane extractive (%*w*/*w*)	0.94
Ethanol extractive content (%*w*/*w*)	1.51
Water extractive content (%*w*/*w*)	10.57
Loss on drying (%*v*/*w*)	9.58
Total ash (%*w*/*w*)	2.54
Acid-insoluble ash (%*w*/*w*)	0.90
Chemical composition	saponin, terpenoids

**Table 2:** Effects of ELJ extract, prednisolone, and aspirin on transudative weight, granuloma weight, and percentage of granuloma inhibition on cotton-pellet-induced granuloma formation in rats.

Groups	Dose (mg/kg)	Granuloma Wet Weight (mg)	Granuloma Dry Weight (mg)	Transudative Weight (mg)	Granuloma Weight (mg/mg Cotton)	GI (%)
Control	-	450.0 ± 27.0	81.8 ± 4.4	368.2 ± 23.4	3.1 ± 0.2	-
Prednisolone	5	291.0 ± 12.4 *	58.6 ± 2.4 *	232.5 ± 10.9 *	1.9 ± 0.1 *	37
Aspirin	300	415.8 ± 33.1	79.5 ± 4.9	336.3 ± 30.1	3.0 ± 0.2	4
ELJ extract	1200	347.4 ± 25.6 *	66.2 ± 3.0 *	281.2 ± 21.7	2.3 ± 0.3	25

Values are expressed as mean ± SEM (*n* = 6). Control: treated with acetone. * Significantly different from its control group, *p* > 0.05.

**Table 3:** Effects of ELJ extract, prednisolone, and aspirin on dry thymus weight with reference to cotton-pellet-induced granuloma formation in rats.

Groups	Dose (mg/kg)	Body Weight (g)			Dry Thymus Weight (mg/100 g)
		Initial	Final	Gain	
Control	-	387.50 ± 13.34	394.17 ± 11.06	6.67 ± 3.07	21.91 ± 1.97
Prednisolone	5	365.00 ± 9.83	353.33 ± 10.14 *	−11.67 ± 6.54 *	17.24 ± 1.79
Aspirin	300	360.83 ± 12.74	361.67 ± 11.38 *	0.83 ± 4.36	16.96 ± 1.27
ELJ extract	1200	383.33 ± 3.80	380.00 ± 5.16	−3.33 ± 4.22	16.99 ± 1.82

Values are expressed as mean ± SEM (*n* = 6). Control: treated with acetone. * Significantly different from its control group, *p* > 0.05.

**Table 4:** Effects of ELJ extract, prednisolone, and aspirin on dry thymus weight with reference to cotton-pellet-induced granuloma formation in rats.

Groups	Dose	Early Phase		Late Phase	
	(mg/kg)	Licking Time (s)	% Inhibition of Licking Response	Licking Time (s)	% Inhibition of Licking Response
Control	-	67.8 ± 4.5	-	93.8 ± 7.1	-
Aspirin	300	45.0 ± 5.3 *	34	0.0 ± 0.0 *	100
Morphine	10	0.0 ± 0.0 *	100	1.5 ± 1.5 *	98
ELJ extract	300	44.8 ± 5.0 *	34	11.2 ± 9.5 *	88
	600	41.8 ± 3.3 *	38	4.8 ± 3.4 *	95
	1200	38.7 ± 3.2 *	43	3.2 ± 3.2 *	97

Values are expressed as mean ± SEM (*n* = 6). Control: treated with acetone. * Significantly different from its control group, *p* > 0.05.

**Table 5:** Effects of ELJ extracts and aspirin on yeast-induced hyperthermia in rats.

Groups	Dose (mg/kg)	Rectal Temperature (°C)					
		Baseline	18 h after Yeast Injection	Time after Drug Administration (min)			
				30 min	60 min	90 min	120 min
Control	-	37.98 ± 0.22	39.20 ± 0.23	39.17 ± 0.30	39.03 ±0.23	39.08 ± 0.21	39.03 ± 0.22
Aspirin	300	38.03 ± 0.21	39.07 ± 0.20	38.30 ± 0.25 *	37.98 ± 0.22 *	37.87 ± 0.18 *	37.75 ± 0.20 *
ELJ extract	300	38.03 ± 0.23	39.23 ± 0.09	39.02 ± 0.15	38.85 ± 0.17	38.85 ± 0.17	38.83 ± 0.20
	600	37.88 ± 0.16	39.23 ± 0.10	38.83 ± 0.20	38.60 ± 0.13	38.65 ± 0.10	38.73 ± 0.07
	1200	38.07 ± 0.15	39.00 ± 0.13	38.30 ± 0.27 *	38.18 ± 0.25 *	38.27 ± 0.27 *	38.08 ± 0.31 *

Values are expressed as mean ± SEM (*n* = 6). Control: treated with acetone. * Significantly different from its control group, *p* > 0.05.

**Table 6:** Effects of ELJ extract, control, and cimetidine in the pylorus ligation rat model.

Group	Gastric Volume (mL)	Secretory Rate (mL/100 g/5 h)	pH	Total Acidity (mEq/100 g/5 h)
Control	3.25 ± 0.35	1.54 ± 0.19	1.95 ± 0.32	29.20 ± 2.65
Cimetidine 100 mg/kg	2.37 ± 0.16	0.87 ± 0.05 *	5.06 ± 0.79 *	13.30 ± 2.35 *
ELJ extract 150 mg/kg	3.43 ± 0.39	1.18 ± 0.17	1.87 ± 0.10	27.02 ± 4.83
ELJ extract 300 mg/kg	3.05 ± 0.43	1.07 ± 0.13	2.31 ± 0.28	27.42 ± 3.25
ELJ extract 600 mg/kg	3.38 ± 0.24	1.26 ± 0.15	2.03 ± 0.42	34.76 ± 5.61

All values are mean ± S.E.M. (*n* = 6). * Significantly different from the control group, *p* < 0.05.

## Data Availability

Not applicable.

## Supplementary Materials

The following supporting information can be downloaded at: https://www.mdpi.com/article/10.3390/life13071465/s1, Table S1: Effects of ELJ extract and phenylbutazone on EPP-induced ear edema in rats.; Table S2: Effects of ELJ extracts and aspirin on carrageenan-induced hind paw edema in rats.; Table S3: Effects of ELJ extract, control, and cimetidine in a rat model of EtOH/HCl-acid-induced gastric lesions.; Table S4: Effect of ELJ and cimetidine on indomethacin-induced gastric ulcer in rat.; Table S5: Effects of ELJ extract, control, and cimetidine in a rat model of the restrained water immersion stress-induced gastric lesions.

## References

[1] Schmid-Schönbein G.W. (2006). Analysis of inflammation. Annu. Rev. Biomed. Eng..

[2] Sostres C., Gargallo C.J., Arroyo M.T., Lanas A. (2010). Adverse effects of non-steroidal anti-inflammatory drugs (NSAIDs, aspirin and coxibs) on upper gastrointestinal tract. Best Pract. Res. Clin. Gastroenterol..

[3] Cui J., Jia J. (2021). Natural COX-2 Inhibitors as Promising Anti-inflammatory Agents: An Update. Curr. Med. Chem..

[4] Marinaccio L., Zengin G., Pieretti S., Minosi P., Szucs E., Benyhe S., Novellino E., Masci D., Stefanucci A., Mollica A. (2023). Food-inspired peptides from spinach Rubisco endowed with antioxidant, antinociceptive and anti-inflammatory properties. Food Chem..

[5] Mollica A., Zengin G., Sinan K.I., Marletta M., Pieretti S., Stefanucci A., Etienne O.K., Jekő J., Cziáky Z., Bahadori M.B. (2022). A Study on Chemical Characterization and Biological Abilities of Alstonia boonei Extracts Obtained by Different Techniques. Antioxidants.

[6] Rehman S.U., Choe K., Yoo H.H. (2016). Review on a Traditional Herbal Medicine, Eurycoma longifolia Jack (Tongkat Ali): Its Traditional Uses, Chemistry, Evidence-Based Pharmacology and Toxicology. Molecules.

[7] Kuo P.-C., Damu A.G., Lee K.-H., Wu T.-S. (2004). Cytotoxic and antimalarial constituents from the roots of Eurycoma longifolia. Bioorg. Med. Chem..

[8] Herbal Medicine Reference Textbook Preparation Subcommittee (2018). Monograph of selected Thai materia medica: PLA LAI PUEAK-RAK. J. Thai Trad. Alt. Med..

[9] Office of Resource and Information Technology Kamphaeng Phet Rajabhat University PLA LAI PUEAK. https://arit.kpru.ac.th/ap2/local/?nu=pages&page_id=1660&code_db=610010&code_type=01.

[10] Bedir E., Abou-Gazar H., Ngwendson J.N., Khan I.A. (2003). Eurycomaoside: A new quassinoid-type glycoside from the roots of Eurycoma longifolia. Chem. Pharm. Bull..

[11] Guo Z., Vangapandu S., Sindelar R.W., Walker L.A., Sindelar R.D. (2005). Biologically active quassinoids and their chemistry: Potential leads for drug design. Curr. Med. Chem..

[12] Ang H.H., Cheang H.S., Yusof A.P.M. (2000). Effects of Eurycoma longifolia Jack (Tongkat Ali) on the initiation of sexual performance of inexperienced castrated male rats. Exp. Anim..

[13] Low B.S., Choi S.B., Abdul Wahab H., Das P.K., Chan K.L. (2013). Eurycomanone, the major quassinoid in Eurycoma longifolia root extract increases spermatogenesis by inhibiting the activity of phosphodiesterase and aromatase in steroidogenesis. J Ethnopharmacol..

[14] Bhat R., Karim A.A. (2010). Tongkat Ali (Eurycoma longifolia Jack): A review on its ethnobotany and pharmacological importance. Fitoterapia.

[15] Han Y.M., Woo S.U., Choi M.S., Park Y.N., Kim S.H., Yim H., Yoo H.H. (2016). Antiinflammatory and analgesic effects of Eurycoma longifolia extracts. Arch. Pharm. Res..

[16] Sireeratawong S., Khonsung P., Piyabhan P., Nanna U., Soonthornchareonnon N., Jaijoy K. (2012). Anti-inflammatory and anti-ulcerogenic activities of Chantaleela recipe. Afr. J. Tradit. Complement. Altern. Med..

[17] Qodriyah H., Asmadi A. (2013). Eurycoma longifolia in Radix for the treatment of ethanol-induced gastric lesion in rats. Pak. J. Biol. Sci..

[18] Bureau of Drug and Narcotic, Department of Medicine Sciences, Ministry of Public Health (2014). Thai Herbal Pharmacopoeia Volume IV.

[19] Tung N.H., Uto T., Hai N.T., Li G., Shoyama Y. (2017). Quassinoids from the root of Eurycoma longifolia and their antiproliferative activity on human cancer cell lines. Pharmacogn. Mag..

[20] Bao Y., Ji W.H., Ma Y.H., Ji L.J. (2006). Simultaneous determination of six main constituents in Swertia of Qinghai Province and Sichuan Province by HPLC. Zhongguo Zhong Yao Za Zhi.

[21] Nie Y., Lin P. (2010). Determination of five active constituents in aerial part of Tibetan medicine Gentiana straminea by HPLC. Zhongguo Zhong Yao Za Zhi.

[22] Brattsand R., Thalén A., Roempke K., Källström L., Gruvstad E. (1982). Influence of 16 alpha, 17 alpha-acetal substitution and steroid nucleus fluorination on the topical to systemic activity ratio of glucocorticoids. J. Steroid. Biochem..

[23] Winter C.A., Risley E.A., Nuss G.W. (1962). Carrageenin-induced edema in hind paw of the rat as an assay for antiiflammatory drugs. Proc. Soc. Exp. Biol. Med..

[24] Swingle K.F., Shideman F.E. (1972). Phases of the inflammatory response to subcutaneous implantation of a cotton pellet and their modification by certain anti-inflammatory agents. J. Pharmacol. Exp. Ther..

[25] Hunskaar S., Hole K. (1987). The formalin test in mice: Dissociation between inflammatory and non-inflammatory pain. Pain.

[26] Teotino U.M., Friz L.P., Gandini A., Dellabella D. (1963). Thio Derivatives of 2,3-Dihydro-4h-1,3-Benzoxazin-4-One. Synthesis And Pharmacological Properties. J. Med. Chem..

[27] Mizui T., Doteuchi M. (1983). Effect of polyamines on acidified ethanol-induced gastric lesions in rats. Jpn. J. Clin. Pharmacol. Ther..

[28] Djahanguiri B. (1969). The production of acute gastric ulceration by indomethacin in the rats. Scand J. Gastroenterol..

[29] Hayden L.J., Thomas G., West G. (1978). Inhibitors of gastric lesions in the rat. J. Pharm. Pharmacol..

[30] Kim J.-H., Kim B.-W., Kwon H.-J., Nam S.-W. (2011). Curative effect of selenium against indomethacin-induced gastric ulcers in rats. J. Microbiol. Biotechnol..

[31] Takagi K., Kasuya Y., Watanabe K. (1964). Studies on the Drugs for Peptic Ulcer. A Reliable Method for Producing Stress Ulcer in Rats. Chem. Pharm. Bull..

[32] Lu C.-L., Li Z.-P., Zhu J.-P., Zhao D.-Q., Ai H.-B. (2011). Studies on functional connections between the supraoptic nucleus and the stomach in rats. J. Physiol. Sci..

[33] SHAY H. (1945). A simple method for the uniform production of gastric ulceration in the rats. Gastroenterology.

[34] Chaingam J., Juengwatanatrakul T., Yusakul G., Kanchanapoom T., Putalun W. (2021). HPLC-UV-Based Simultaneous Determination of Canthin-6-One Alkaloids, Quassinoids, and Scopoletin: The Active Ingredients in Eurycoma Longifolia Jack and Eurycoma Harmandiana Pierre, and Their Anti-Inflammatory Activities. J. AOAC Int..

[35] Miyake K., Tezuka Y., Awale S., Li F., Kadota S. (2009). Quassinoids from Eurycoma longifolia. J. Nat. Prod..

[36] George V.C., Dellaire G., Rupasinghe H.V. (2017). Plant flavonoids in cancer chemoprevention: Role in genome stability. J. Nutr. Biochem..

[37] Cicero A.F., Allkanjari O., Busetto G.M., Cai T., Larganà G., Magri V., Perletti G., Della Cuna F.S.R., Russo G.I., Stamatiou K. (2019). Nutraceutical treatment and prevention of benign prostatic hyperplasia and prostate cancer. Arch. Ital. Urol. Androl..

[38] Majidi Wizneh F., Zaini Asmawi M. (2014). Eurycoma longifolia Jack (*Simarubaceae*); Advances in Its Medicinal Potentials. Pharmacogn. J..

[39] Pandey A., Tripathi S. (2014). Concept of standardization, extraction and pre phytochemical screening strategies for herbal drug. J Pharmacogn. Phytochem..

[40] Dhawan D., Gupta J. (2017). Research article comparison of different solvents for phytochemical extraction potential from datura metel plant leaves. Int. J. Biol. Chem..

[41] Truong D.H., Nguyen D.H., Ta N.T.A., Bui A.V., Do T.H., Nguyen H.C. (2019). Evaluation of the Use of Different Solvents for Phytochemical Constituents, Antioxidants, and In Vitro Anti-Inflammatory Activities of *Severinia buxifolia*. J. Food Qual..

[42] Huang Y.-P., Chang J.I. (2010). Biodiesel production from residual oils recovered from spent bleaching earth. Rene. Energ..

[43] Hou W., Xiao X., Guo W., Zhang T. (2011). Advances in Studies on Chemistry, Pharmacological Effect, and Pharmacokinetics of Eurycoma longifolia. Chin. Herb. Med..

[44] Varghese C.P., Ambrose C., Jin S., Lim Y., Keisaban T. (2013). Antioxidant and anti-inflammatory activity of Eurycoma longifolia Jack, a traditional medicinal plant in Malaysia. Int. J. Pharm. Sci. Nanotech..

[45] Kunle O.F., Egharevba H.O., Ahmadu P.O. (2012). Standardization of herbal medicines-A review. Int. J. Biodivers. Conserv..

[46] Tada H., Yasuda F., Otani K., Doteuchi M., Ishihara Y., Shiro M. (1991). New antiulcer quassinoids from Eurycoma longifolia. Eur. J. Med. Chem..

[47] Hajjouli S., Chateauvieux S., Teiten M.H., Orlikova B., Schumacher M., Dicato M., Choo C.Y., Diederich M. (2014). Eurycomanone and eurycomanol from Eurycoma longifolia Jack as regulators of signaling pathways involved in proliferation, cell death and inflammation. Molecules.

[48] Tran T.V.A., Malainer C., Schwaiger S., Atanasov A.G., Heiss E.H., Dirsch V.M., Stuppner H. (2014). NF-κB Inhibitors from Eurycoma longifolia. J. Nat. Prod..

[49] Solomon M., Erasmus N., Henkel R. (2014). In vivo effects of Eurycoma longifolia Jack (Tongkat Ali) extract on reproductive functions in the rat. Andrologia.

[50] Ang H.H., Cheang H.S. (2001). Effects of Eurycoma longifolia jack on laevator ani muscle in both uncastrated and testosterone-stimulated castrated intact male rats. Arch. Pharm. Res..

[51] Ang H.H., Sim M.K. (1998). Eurycoma longifolia JACK and orientation activities in sexually experienced male rats. Biol. Pharm. Bull..

[52] Lee S.P., Tasman-Jones C. (1978). Prevention of acute gastric ulceration in the rat by cimetidine, a histamine H2-receptor antagonist. Clin. Exp. Pharmacol. Physiol..

[53] Turck D., Bohn T., Castenmiller J., De Henauw S., Hirsch-Ernst K.I., Maciuk A., Mangelsdorf I., McArdle H.J., Naska A., Pelaez C. (2021). Safety of Eurycoma longifolia (Tongkat Ali) root extract as a novel food pursuant to Regulation (EU) 2015/2283. EFSA J..

[54] Carlson R.P., O’Neill-Davis L., Chang J., Lewis A.J. (1985). Modulation of mouse ear edema by cyclooxygenase and lipoxygenase inhibitors and other pharmacologic agents. Agents Actions.

[55] Rubin R., Strayer D.S., Rubin E. (2008). Rubin’s Pathology: Clinicopathologic Foundations of Medicine.

[56] Borges R.S., Palheta I.C., Ota S.S.B., Morais R.B., Barros V.A., Ramos R.S., Silva R.C., Costa J.D.S., Silva C., Campos J.M. (2019). Toward of Safer Phenylbutazone Derivatives by Exploration of Toxicity Mechanism. Molecules.

[57] Patil K.R., Mahajan U.B., Unger B.S., Goyal S.N., Belemkar S., Surana S.J., Ojha S., Patil C.R. (2019). Animal models of inflammation for screening of anti-inflammatory drugs: Implications for the discovery and development of phytopharmaceuticals. Int. J. Mol. Sci..

[58] Di Rosa M. (1972). Biological properties of carrageenan. J. Pharm. Pharmacol..

[59] Rosa S.G., Brüning C.A., Pesarico A.P., de Souza A.C.G., Nogueira C.W. (2018). Anti-inflammatory and antinociceptive effects of 2, 2-dipyridyl diselenide through reduction of inducible nitric oxide synthase, nuclear factor-kappa B and c-Jun N-terminal kinase phosphorylation levels in the mouse spinal cord. J. Trace. Elem. Med. Biol..

[60] Sarraf P., Sneller M.C. (2005). Pathogenesis of Wegener’s granulomatosis: Current concepts. Expert Rev. Mol. Med..

[61] Katzung B.G., Trevor A. (2007). Chapter 36: Nonsteroidal Anti-Inflammatory Drugs, Disease-Modifying Antirheumatic Drugs, Nonopioid Analgesics, & Drugs Used in Gout. Basic & Clinical Pharmacology.

[62] Tjølsen A., Berge O.G., Hunskaar S., Rosland J.H., Hole K. (1992). The formalin test: An evaluation of the method. Pain.

[63] Devi B.P., Boominathan R., Mandal S.C. (2003). Evaluation of antipyretic potential of Cleome viscosa Linn. (Capparidaceae) extract in rats. J. Ethnopharmacol..

[64] Moltz H. (1993). Fever: Causes and consequences. Neurosci. Biobehav. Rev..

[65] Lovejoy F.H. (1978). Aspirin and acetaminophen: A comparative view of their antipyretic and analgesic activity. Pediatrics.

[66] Rawlins M., Postgrad R. Mechanism of salicylate-induced antipyresis. Proceedings of the Pharmacology Thermoregulatory Proceeding Satellite Symposium.

[67] Szelenyi I., Brune K. (1988). Possible role of oxygen free radicals in ethanol-induced gastric mucosal damage in rats. Dig. Dis. Sci..

[68] Guslandi M. (1987). Effects of ethanol on the gastric mucosa. Dig. Dis..

[69] Cho C.H., Ogle C.W. (1992). The pharmacological differences and similarities between stress-and ethanol-induced gastric mucosal damage. Life Sci..

[70] Drini M. (2017). Peptic ulcer disease and non-steroidal anti-inflammatory drugs. Aust. Prescr..

[71] Wallace J.L., McKnight W., Reuter B.K., Vergnolle N. (2000). NSAID-induced gastric damage in rats: Requirement for inhibition of both cyclooxygenase 1 and 2. Gastroenterol.

[72] Guo S., Gao Q., Jiao Q., Hao W., Gao X., Cao J.-M. (2012). Gastric mucosal damage in water immersion stress: Mechanism and prevention with GHRP-6. World. J. Gastroenterol..

[73] Simões S., Lopes R., Campos M.C.D., Marruz M.J., da Cruz M.E.M., Corvo L. (2019). Animal models of acute gastric mucosal injury: Macroscopic and microscopic evaluation. AMEM.

[74] Adinortey M.B., Ansah C., Galyuon I., Nyarko A. (2013). In vivo models used for evaluation of potential antigastroduodenal ulcer agents. Ulcers.

[75] Jayachitra C., Jamuna S., Ali M.A., Paulsamy S., Al-Hemaid F.M. (2018). Evaluation of traditional medicinal plant, Cissus setosa Roxb.(*Vitaceae*) for antiulcer property. Saudi J. Biol. Sci..

[76] Mahattanadul S., Ridtitid W., Nima S., Phdoongsombut N., Ratanasuwon P., Kasiwong S. (2011). Effects of Morinda citrifolia aqueous fruit extract and its biomarker scopoletin on reflux esophagitis and gastric ulcer in rats. J. Ethnopharmacol..

[77] George A., Suzuki N., Abas A.B., Mohri K., Utsuyama M., Hirokawa K., Takara T. (2016). Immunomodulation in Middle-Aged Humans Via the Ingestion of Physta^®^ Standardized Root Water Extract of Eurycoma longifolia Jack—A Randomized, Double-Blind, Placebo-Controlled, Parallel Study. Phytother. Res..

