# Supplementary material for: Anti-Inflammatory, Antinociceptive, Antipyretic, and Gastroprotective Effects of Eurycoma longifolia Jack Ethanolic Extract
Source: Life (Basel). 2023 Jun 28;13(7):1465. doi: 10.3390/life13071465 (PMC10381342; doi:10.3390/life13071465)
Supplement: Supplementary file 1 [file life-13-01465-s001.zip › life-2418833-supplementary.pdf]

life

MDPI

Supplementary Materials

Anti-Inflammatory, Antinociceptive, Antipyretic, and Gastroprotective Effects of *Eurycoma longifolia* Jack Ethanolic Extract

Supplementary Table S1. Effects of ELJ extract and phenylbutazone on EPP-induced ear edema in rats.

Group	Dose (mg/ear)	Edema thickness (µm)				Edema inhibition (%)			
		15 min	30 min	60 min	120 min	15 min	30 min	60 min	120 min
Control	-	110 ± 9	232 ± 23	338 ± 27	230 ± 15	-	-	-	-
Phenylbutazone	1	42 ± 10*	93 ± 10*	178 ± 14*	132 ± 10*	62	60	47	43
ELJ extract	1	45 ± 9*	123 ± 27*	153 ± 39*	125 ± 38*	59	47	55	46
	2	37 ± 7*	93 ± 17*	137 ± 26*	123 ± 30*	67	60	60	46
	4	27 ± 7*	78 ± 7*	120 ± 33*	95 ± 20*	76	66	64	59

Values are expressed as mean ± SEM (n = 6). Control: treated with acetone. * Significantly different from its control group, $p > 0.05$.

Supplementary Table S2. Effects of ELJ extracts and aspirin on carrageenan-induced hind paw edema in rats.

Groups	Dose (mg/kg)	Time after 1% carrageenin injection					
		1 hour		3 hours		5 hours	
		Edema volume (ml)	% Inhibition	Edema volume (ml)	% Inhibition	Edema volume (ml)	% Inhibition
Control	-	0.41 ± 0.07	-	1.28 ± 0.11	-	1.18 ± 0.04	-
Aspirin	300	0.17 ± 0.06*	58	0.64 ± 0.12*	50	0.61 ± 0.10*	48
ELJ extract	300	0.35 ± 0.14	15	1.25 ± 0.09	2	1.01 ± 0.17	9
	600	0.26 ± 0.06	36	1.13 ± 0.06	12	1.00 ± 0.06	15
	1200	0.14 ± 0.05*	66	0.87 ± 0.11*	32	0.88 ± 0.09*	25

Values are expressed as mean ± SEM (n = 6). Control: treated with acetone. * Significantly different from its control group, $p > 0.05$.

Supplementary Table S3. Effects of ELJ extract, control, and cimetidine in a rat model of EtOH/HCl acid-induced gastric lesions.

Group	Dose (mg/kg)	Ulcer Index[1]	% inhibition
Control	-	81.52 ± 7.12	-
Cimetidine	100	20.02 ± 3.48*	75
ELJ extract	150	36.92 ± 5.27*,#	55
ELJ extract	300	32.38 ± 6.92*,#	60
ELJ extract	600	30.50 ± 2.36*,#	62

[1] Ulcer indexes are mean ± S.E.M. (N=6). * Significantly different from the control group, $p < 0.05$ and # Significant different from the cimetidine group, $p < 0.05$.

Supplementary Table S4. Effect of ELJ and cimetidine on indomethacin-induced gastric ulcer in rat.

Group	Dose (mg/kg)	Ulcer Index[1]	% inhibition
Control	-	9.30 ± 0.59	-
Cimetidine	100	0.40 ± 0.27*	95
ELJ extract	150	7.20 ± 1.16*,#	23
ELJ extract	300	1.12 ± 0.31*	88
ELJ extract	600	0.65 ± 0.36*	93

[1] Ulcer indexes are mean ± S.E.M. (n=6). * Significantly different from the control group, $p < 0.05$ and # Significant different from the cimetidine group, $p < 0.05$.

Supplementary Table S5. Effects of ELJ extract, control, and cimetidine in a rat model of the restraint water immersion stress-induced gastric lesions.

Group	Dose (mg/kg)	Ulcer Index[1]	% inhibition
Control	-	10.42 ± 2.36	-
Cimetidine	100	0.80 ± 0.28*	92
ELJ extract	150	5.08 ± 0.96*,#	51
ELJ extract	300	1.95 ± 0.44*	81
ELJ extract	600	1.82 ± 0.41*	82

[1] Ulcer indexes are mean ± S.E.M. (*n*=6). * Significantly different from the control group, $p < 0.05$ and # Significant different from the cimetidine group, $p < 0.05$.
